# Treating alcohol use disorder in the absence of specialized services – evaluation of the moving inpatient *Treatment Camp* approach in Uganda

**DOI:** 10.1186/s12888-021-03593-5

**Published:** 2021-12-01

**Authors:** Verena Ertl, Melissa Groß, Samuel Okidi Mwaka, Frank Neuner

**Affiliations:** 1 Clinical Psychology and Biopsychology, Department of Psychology, Catholic University Eichstätt-Ingolstadt, Ostenstraße 25, 85072 Eichstätt, Germany; 2 vivo international (www.vivo.org), Konstanz, Germany; 3 Clinical Psychology and Psychotherapy, Department of Psychology, Bielefeld University, Universitätsstraße 25, 33615 Bielefeld, Germany; 4 Program for Prevention, Awareness, Counseling and Treatment of Alcoholism (PACTA; www.pactaguluganda.org.ug), Plot 1 Burcoro Road, Wiaworanga, Gulu, Uganda

**Keywords:** Alcohol use disorder, Addiction, Treatment, Withdrawal, Detoxification, Family violence, Intimate partner violence, Low and middle income country, Treatment cost, Service user involvement

## Abstract

**Background:**

The gap between service need and service provision for alcohol-related disorders is highest in resource-poor countries. However, in some of these contexts, local initiatives have developed pragmatic interventions that can be carried out with limited specialized personnel. In an uncontrolled treatment study, we aimed to evaluate the feasibility, acceptability, safety, costs and potential effects of an innovative locally developed community-based program (the *Treatment Camp*) that is based on an inpatient clinic that moves from community to community.

**Methods:**

Out of 32 treatment-seeking individuals 25 took part in the one-week *Treatment Camp* that included detoxification and counseling components. Re-assessments took place 5 and 12 months after their participation. We explored the course of a wide range of alcohol-related indicators, using the Alcohol Use Disorders Identification Test (AUDIT) as primary outcome complemented by a timeline follow-back approach and the Obsessive Compulsive Drinking Scale. Additionally, we assessed impaired functioning, alcohol-related stigmatization, symptoms of common mental health disorders and indicators of family functioning as reported by participants’ wives and children.

**Results:**

All alcohol-related measures decreased significantly after the *Treatment Camp* and remained stable up to the 12-month-assessment with high effect sizes ranging from 0.89 to 3.49 (Hedges’s *g*). Although 92% of the participants had lapsed at least once during the follow-up period, 67% classified below the usually applied AUDIT cutoff for hazardous drinking (≥ 8) and no one qualified for the dependent range (≥ 20) one year after treatment. Most secondary outcomes including impaired functioning, alcohol-related stigmatization, symptoms of depression and indicators of family functioning followed the same trajectory.

**Conclusions:**

We found the *Treatment Camp* approach to be acceptable, feasible, safe and affordable (approx. 111 USD/patient) and we could obtain preliminary evidence of its efficacy. Due to its creative combination of inpatient treatment and monitoring by medical personnel with local mobility, the *Treatment Camp* appears to be more accessible and inclusive than other promising interventions for alcohol dependent individuals in resource-poor contexts. Effects of the approach seem to extend to interactions within families, including a reduction of dysfunctional and violent interactions.

**Supplementary Information:**

The online version contains supplementary material available at 10.1186/s12888-021-03593-5.

Guidelines for the treatment of alcohol dependence recommend assisted withdrawal, either offered community-based or residential. Assisted withdrawal programs should include a drug regimen and psychological interventions including individual and group treatments and psychoeducational content. Assistance to attend mutual-help groups and family and career support should complete withdrawal interventions. Residential assisted withdrawal is especially recommended if patients belong to vulnerable groups, e.g. those having psychiatric or physical comorbidities. In residential care the use of benzodiazepines complemented by symptom-tailored drugs in case of complications, including states of abstinence deliria and withdrawal-related seizures, is recommended. In case a fixed-dose regimen is implemented, it should be reduced to zero over a period of 7–10 days. Interventions after the detoxification phase include individual psychological interventions like (cognitive) behavioral therapies as well as social network and environment-based therapies, all focusing on alcohol and relapse prevention. Pharmacological intervention may also be considered after assisted withdrawal to further support abstinence. Finally, guidelines recommend care coordination and case management in the treatment of people with moderate to severe alcohol dependence [1–3].

While these recommendations are consensus among health care and rehabilitation providers worldwide, the reality of service provision is different for populations in low and middle income countries (LMIC), and especially for conflict-affected and displaced populations (e.g. [4–15]). Since 2008, World Health Organization’s (WHO) Mental Health Gap Action Program (mhGAP) draws attention to the mismatch between the prevalence of alcohol-related disorders and the number of those receiving treatment in low-resource contexts. Despite the undisputed burden of an alcohol use disorder (AUD) for the individuals, families and communities, the treatment gap is high, even in comparison with other mental disorders. Globally, about 78% of affected individuals remain untreated, most of them residing in LMIC [16]. The United Nations (UN) sustainable development goals specifically mention alcohol under goal three that is dealing with good health and well-being. The aspiration is to strengthen the prevention and treatment of substance use, including harmful use of alcohol [17]. Despite WHO’s and UN’s calls for action, it seems there has been hardly any progress, since more recent literature focusing on low-resource settings still estimated an AUD lifetime treatment gap of at least 87% [15, 18].

At the same time, AUD are frequent and have more detrimental consequences in LMIC compared to high-income settings [19–22]. Excessive alcohol use and dependency are generally linked to negative individual, familial, and societal consequences like premature illness and death, loss of functionality and productivity, domestic and community violence, social decline and stigmatization and delinquency [21, 23–29]. The negative effects of excessive alcohol consumption are aggravated in LMIC by the use of unsafe home-made alcohol, and the rapid existential threat to the whole family resulting from the costs of consumption. Moreover, against the background of high rates of early childhood and conflict-related violence, of poverty and unemployment, alcohol consumption must be understood as self-medication considering comorbid psychopathology, as short-term problem solver, as diversion and as a driving element in cycles of violence [5, 24, 25, 30–32].

According to the current global status report on alcohol and health [19], Uganda is among the top 10 African countries in per capita consumption. Due to a high number of abstainers, especially among women, the average (9.5 l of pure alcohol being consumed per year by Ugandans ≥15 years) seems quite low, while the total alcohol per capita consumption in male drinkers (≥ 15 years) is exceptionally high, with 32.7 l of pure alcohol annually. Worldwide, consumers drink about 15.1 l. The African region ranges more than 20% above this global average (18.4 l). The global status report on alcohol and health [19] reports alcohol dependence in 4.2% of the Ugandan male population (12-month prevalence estimates), whereas another countrywide survey reports 9.8% using the same timeframe [33]. In the formerly conflict-affected northern areas, studies found similar levels of dependent drinking at 9.9 and 8.9%, respectively, with both studies reporting 12-month prevalence rates for males estimated by AUDIT scores of 20 and above [5, 6]. Compared to the global status report on alcohol and health [19], this may hint at elevated numbers related to the past conflict and its consequences. Taken together, prevalence data suggests that those (predominantly male) Ugandans who drink have especially harmful drinking habits and patterns and this seems to be true for many LMIC [19].

Specialized services for moderate and severe alcohol addiction that are able to provide drug regimen and constant monitoring of the medical status are scarce in these contexts [8]. Therefore, the mere distance to providers prohibits many affected individuals from service use. Additionally, specialized health care is not affordable for the majority of dependent drinkers in LMIC. A lack of awareness and education about alcohol-related disorders and their treatability as well as the fear of stigmatization are further contributing factors [14, 16, 34]. The majority of alcohol-related programs that have been scientifically evaluated in LMIC use brief interventions and target hazardous and harmful alcohol users. As a consequence, most of the current prevention and intervention research either excludes severely addicted individuals or doesn’t differentiate between hazardous and harmful alcohol use and dependence (cf. a recent systematic review reporting on the effectiveness of psychosocial interventions for hazardous and harmful alcohol use in LMIC [11]). This is surprising, since special additional care is warranted for individuals with severe alcohol use disorder (AUD). The involvement of medical and psychiatric experts is key to manage withdrawal symptoms, including potentially dangerous developments, like deliria. Additionally, the numerous physical comorbidities from malnutrition to liver problems need to be addressed professionally when dealing with severe addiction in LMIC.

Publications scientifically addressing the evaluation of withdrawal interventions in LMIC are scarce. A recent exception being Nadkarni et al. [35], who piloted a lay counselor-delivered home-based detoxification and relapse prevention program in Goa, India. In the absence of clinics and secondary or tertiary care facilities, or them being unreachable and unaffordable for most AUD patients, task-shifting to lay workers and community-based service provision seems the only practicable solution. However, in the case of the exploratory study by Nadkarni et al. [35] and all other community-based detoxification programs (cf. a review by Nadkarni et al. [14]) contraindications for home-detoxification were numerous and likely to apply to a significant number of AUD patients in LMIC. Amongst others, these were a lifetime history of seizures, unexplained loss of consciousness, medical conditions (e.g. heart disease) or current conditions, such as mental health problems (psychoses, suicidality, hallucinations, depression, consuming other substances (except tobacco)) and current physical health problems from uncontrolled hypertension to liver compromise (for a full list of typical contraindications see Nadkarni et al. [14, 35]).

Northern Uganda is an example of an area affected by all the structural and sociocultural barriers to care that have been mentioned above. In light of these restrictions, formerly AUD-affected individuals, their family members and concerned volunteers founded a community-based nongovernmental organization (NGO) dealing with addiction, PACTA (Program for Prevention, Awareness, Counseling and Treatment of Alcoholism). In LMIC it is not unusual for NGOs to fill gaps in the public health sector. The so-called *Treatment Camp* intervention developed by PACTA tries to overcome the most relevant limitations of usual care for moderate and severe AUD in LMIC. The innovative aspect of the *Treatment Camp* is that instead of establishing a long-term clinical facility in permanent real estate, the program moves around the region and takes place in temporarily rented sites that provide sleeping and teaching facilities on a controllable compound near the target-communities. In this way, a temporary inpatient-setting for withdrawal treatment can be established that is reachable and affordable to most individuals affected by AUD. The *Treatment Camp* is continuously moving from community to community with counselors and medical staff and therefore can cover a wide range of underserved areas, while providing all necessary nutritional, medical and psychological care in one location for a predefined amount of time (usually 7 days). The inpatient nature of the approach allows for a substantial reduction of the ineligibility criteria that have been limiting other community-based detoxification approaches [14, 35].

This study aimed to externally evaluate PACTA’s *Treatment Camp* approach. Next to its impact on alcohol-related measures, we also address issues of acceptability, feasibility and safety. Additionally, if available we interviewed participants’ partners and children to get pre-treatment, and follow-up information on relationship quality and violence at home. Studies evaluating potential changes in family violence after mental health interventions with an individual family member, including alcohol-related interventions, are extremely rare. Tol et al. [36] reviewed the literature for LMIC and found only seven eligible studies. They report that alcohol-focused intervention studies did not show benefits on intimate partner violence (IPV). Our quantitative data collection was complemented by a way of service user involvement unique in this context. We conducted qualitative interviews with participants, which we analyzed using the framework method [37]. Participants were asked a) which elements they perceived as helpful and which as not helpful during the *Treatment Camp*, b) what they perceived as helpful versus not helpful in the time after the *Treatment Camp* and c) what factors they perceived as either causing relapse, or successful abstinence, or the successful stable reduction of alcohol intake to harmless levels. Thereby, we tried to shed light on topics and elements of the intervention and later environmental conditions which facilitated healing and which hindered healing in the eyes of the participants. Qualitative outcomes and partner- and children-data can only be addressed in brief here. Details can be found in the supplementary material.

## Methods

### Participants and procedures

The current study was a cooperation between the community-based NGO PACTA (Program for Prevention, Awareness, Counseling and Treatment of Alcoholism), the NGO vivo international and Bielefeld University. It was a single-armed study examining a convenience sample of alcohol dependent men and women living in Gulu, who were interested in taking part in an inpatient treatment program that was announced via the radio and conducted by PACTA. Personnel of vivo international, an NGO experienced in providing mental health-related services in the area, in cooperation with Bielefeld University served as external evaluators that conducted pre-treatment and follow-up assessments. Evaluators who carried out assessments before and after the intervention phase were fully independent of practitioners and counselors providing the intervention. Due to high rates of illiteracy assessments were conducted in interview format by nine counselors affiliated with the Outpatient Clinic for Survivors of Violence and Trauma and proficient in using all screening instruments in the local language Luo. Clinical psychologists with at least MSc-level education, experienced in cross-cultural research and familiar with the Northern Ugandan context were present at all interview times and provided supervision and training. Pre-treatment interviews took place on the premises of the Outpatient Clinic for Survivors of Violence and Trauma in Gulu, mostly one week before treatment start. Five- and twelve-month follow-up assessments took place either in the same location or - in case it was more convenient for the participant - in a private place at their home. Questionnaires were routinely checked for missing items and inconsistencies on site. Before starting the interview, the project and procedures were explained in detail and participants were encouraged to raise questions. Written informed consent was obtained (signature or fingerprints). Participants did not receive any financial or material benefit for participating in the study except a compensation of 5000 UGX (approximately 1.80 USD) for their transport costs.

**Fig. 1:**
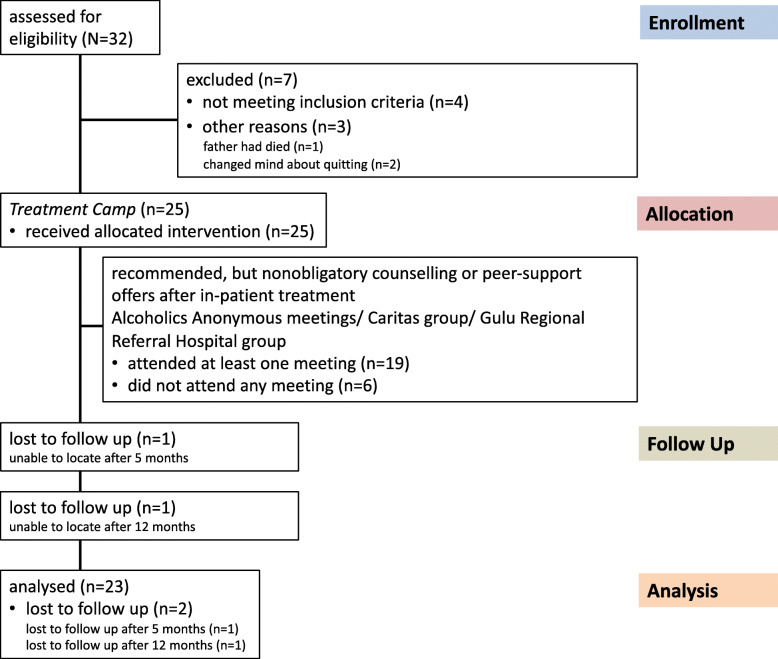
*Flow chart of participants through the trial*

**Fig. 2:**
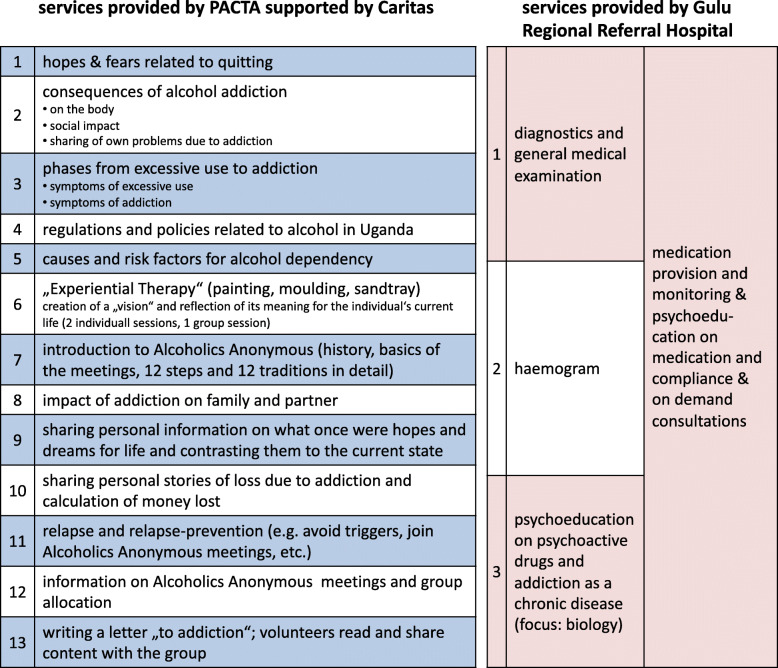
*Treatment components and content by service provider*

In total, 32 potential participants were assessed for treatment eligibility, 7 were excluded. Of those excluded 4 did not meet the inclusion criteria (an AUDIT score of at least 16 and/or showing up at the scheduled pre-treatment interview drunk according to breathalyzer results) and 3 changed their mind about participation. Consequently 25 participants were invited to enter the *Treatment Camp*. Diagnostic status according to the ICD-10 was additionally assessed on entry by the practitioners routinely charged with the task of diagnosing and treating psychiatric patients in the Gulu Regional Referral Hospital (GRRH). Senior Psychiatric Clinical Officers from GRRH supported by a Laboratory Technician conducted the initial medical assessment and reviewed the exclusion criteria. These were contagious diseases (e.g. active TB, Hepatitis B), acute psychosis, severe memory difficulties, epilepsy and severe (chronic) physical conditions requiring immediate medical attention. All 25 positively screened individuals were confirmed as suffering from alcohol dependence and received the allocated intervention components (cf. Fig. 2). At the five-month follow-up we failed to locate one participant. Another participant was not traceable at the 12-month follow-up (cf. Fig. 1). Characteristics of the sample are summarized in Table 1.

**Table 1:** Sociodemographic information (*n* = 25)

Age, mean (SD)	40.60 (10.71)
Male, N (%)	23 (92)
Marital Status, N (%)	
single	8 (32)
married/cohabiting	8 (32)
divorced/separated	7 (28)
widowed	2 (8)
Level of education, N (%)	
no schooling/some primary	4 (16)
completed primary school/vocational school/some secondary	7 (28)
completed secondary school	5 (20)
completed “A-level”/some university	4 (16)
completed university	5 (20)
Regular income, N (%)	14 (56)
Household composition, mean (SD)	
household members	5.84 (3.95)
biological children	2.96 (2.26)
biological children living in household	1.04 (1.59)

Ethical approval for the study was provided by the Ethical Committee of Bielefeld University following the guidelines of the German Psychological Society. These guidelines are in agreement with the American Psychological Association’s code of ethics. All subjects gave written informed consent in accordance with the Declaration of Helsinki.

### Treatment

Staff of PACTA were in charge of preparing, organizing and determining the content of the *Treatment Camp* that was defined as a one-week intensive inpatient detoxification program complemented by psycho-therapeutic content. Since no specialized detoxification and rehabilitation facilities exist in Northern Uganda, venues where usually community meetings are held, or workshop and training halls with accommodation facilities are chosen as locations for the *Treatment Camps* by PACTA. We scientifically studied PACTA’s second *Treatment Camp* held at the Comboni Missionaries’ Animation Centre, Gulu. Concerning psychiatric and medical requirements PACTA was supported by staff from GRRH. Medication used included diazepam, promethazine, carbamazepine, fluoxetine, amitriptyline, haloperidol, the use of antibiotics (cotrimoxazole, amoxicillin and ciprofloxacin), pain medication (paracetamol and diclofenac) and the administration of vitamins. Staff from Caritas Gulu supported PACTA in psychoeducational and motivational sessions. Caritas Gulu is connected to the internationally active aid organization Caritas and amongst other activities provides counseling services as well as training in Northern Uganda. One staff from PACTA was also on site at night to act as contact in case of a crisis. In case of a medical emergency, a referral to the next health unit would have been made. Day one was reserved for arrival, diagnostics, medical examination and treatment by GRRH staff as described in Fig. 2. Days 2 through 6 were filled with interventions by PACTA (supported by Caritas) staff as indicated in Fig. 2 and by medication provision and monitoring through GRRH staff. Day 7 was reserved for validating the effort each participant put into personal healing and farewell. A key part of the treatment content was the introduction of the Alcoholics Anonymous philosophy and the strong recommendation to join AA meetings after the inpatient Camp phase. Alternatively, staff from Caritas and GRRH invited participants to join their open-topic psychosocial groups on a voluntary basis. From the time after the intensive treatment week up to the 12-month follow-up, 19 participants attended at least one of these support group meetings at either Caritas, or GRRH or an AA meeting, whereas 6 did not attend any meeting at all.

### Measures

Luo (local language in northern Uganda) versions of the screening instruments were either already existing or developed for this study [5, 24]. We used a translation and blind back translation procedure create initial versions. Final versions were composed in discussion with expert clinicians and experienced bilingual mental health counselors. Recommendations for cultural adaption, ensuring conceptual, functional and semantic equivalence were considered [38].

#### Sociodemographics

Among others, common sociodemographic variables were recorded for each participant, including year of birth, gender, marital status, level of education, household composition and economic status.

#### Alcohol consumption and symptoms of alcohol use disorder

Alcohol consumption and alcohol-related symptoms were measured via the 10-item interview version of the well-established Alcohol Use Disorders Identification Test (AUDIT [39]). Items 1 to 3 assess frequency and typical quantity of alcohol consumption as well as frequency of heavy drinking. Items 4 to 6 determine symptoms of dependence and items 7 to 10 assess harmful alcohol use. Items 1 to 8 are coded on 5-point scales ranging from 0 to 4 with varying anchor descriptions fitting the content of the respective question. Items 9 and 10 offer only 3 anchors with scoring options 0, 2 and 4. The sum of items 1 through 10 is commonly used for score-interpretation. The AUDIT identifies hazardous and harmful alcohol use and possible dependence being consistent with ICD-10 definitions. A score of 8 to 15 has been established as an indicator for hazardous use, between 16 and 19 as indicating harmful drinking and a score of 20 and above as indicating dependent drinking. The AUDIT has been reported to accurately measure risk across gender, age and cultures [40, 41]. The AUDIT had already been successfully employed in northern Uganda in earlier research [5, 6]. In addition to the AUDIT we asked for age at first time consumption and age at the beginning of dependency. Typical consumption on an ordinary drinking-day was converted to standard drinks. One standard drink was defined to contain 13 g of pure ethanol. Interviewers were trained to use a conversion table we developed for the Ugandan context [5, 31] to be able to translate typical types and serving sizes of local alcoholic beverages, including locally brewed unrecorded alcohol into standard drinks.

#### Lapse and relapse assessment

We used a timeline follow back approach [42, 43] to assess drinking frequency and quantity at both follow-ups. The trained assessors guided the participants with the help of standardized questions and a calendar to memorize their drinking habits as accurately as possible after the end of the *Treatment Camp*. As suggested by Sobell et al. [42] the interviewers first established significant anchor dates. The timeline follow back approach allowed the establishment of several outcome measures, among these days to first lapse or relapse, number of days with consumption, number of days in abstinence, percentage of days consuming and percentage of days in abstinence.

#### Craving

The Obsessive Compulsive Drinking Scale (OCDS) is a 14-item instrument measuring perceived craving in the week prior to assessment and was implemented since craving has been associated with risk of relapse and seems to be a relevant aspect of alcohol dependency beyond alcohol consumption [44–46]. Participants answer each item on a 5-point scale ranging from 0 to 4, representing increasing intensity. The OCDS was designed to assess two dimensions of craving, obsessive alcohol-related thoughts (7 items) and compulsive behaviors toward alcohol (7 items). Consequently, the instrument generally allows for the calculation of two subscale scores and a total score. We followed the algorithm suggested by the authors to calculate subscale and total scores. The scale has been recommended as a tool to measure severity and improvement during alcoholism treatment trials [44, 45]. A specialty of the OCDS is that the items don’t use uniform scaling. During the adaptation process it became evident that the individually formulated five anchors per item that may work well in settings where literate participants fill in the questionnaire by themselves, were too complex to translate and explain in the interview format in the given context. Consequently, we adjusted the scaling slightly by removing each item’s written anchors for the graded options 1, 2 and 3. This means for each item only the extremes of 0 and 4 were defined with written labels. For example, in the original version item one, asking about how much of the time when not drinking is occupied by ideas, thoughts, impulses or images related to drinking, has the anchors 0 (“none”), 1 (“less than 1 hour a day”), 2 (“1-3 hours a day”), 3 (“4-8 hours a day”) and 4 (“greater than 8 hours a day”). In our version the anchors were 0 (“none”), 1, 2, 3, and 4 (“greater than 8 h a day”). Assessors were trained to explain that 1, 2, and 3 should be seen as continuous gradings between the extremes 0 (“none”) and 4 (“greater than 8 h a day”). A further change concerned item 3 that originally combined interference of craving with social or work functioning. Combining the two areas of life “social” and “work” did not fit the Ugandan context. We split the item into two for better cultural fit and understandability.

Additionally, we assessed craving with four items using visual analogue scales ranging from 0 (“not present/never”) to 10 (“very strong/always”) following suggestions in the literature that state that craving has different facets and that the OCDS scores appear to measure aspects that only partly overlap with analogue craving measures [47–49]. The first three items targeted the perceived intensity of craving a) at the very time of the interview, b) averagely during the past seven days and c) the peak intensity of the past seven days. The fourth item assessed frequency of craving in the past seven days. Craving scales in that format have been applied successfully internationally [44, 45, 47, 48].

#### Psychopathology

The Posttraumatic Stress Diagnostic Scale (PDS [50]) was implemented to assess symptoms of PTSD. It provides measures of overall and subscale symptom severity. Its 17 items reflect the core PTSD criteria of reexperiencing, avoidance and hyperarousal according to the DSM IV [51]. Each item can be scored on a 4-point scale ranging from 0 (“not at all or only one time in the past month”) to 3 (“five or more times a week or almost always”). A validation study in Northern Uganda confirmed applicability, very good internal consistency and good correspondence with expert diagnoses of PTSD [52]. When the A-criterion for PTSD was met, we calculated the sum of the 17 symptom-items to obtain a measure of symptom severity.

The 15-item Depression-section of the Hopkins Symptom Checklist (DHSCL [53]) was used to assess the perceived intensity of symptoms of depression in the week prior to the interview. Answers are coded on a 4-point scale ranging from 1 (“the symptom bothered or distressed me not at all”) to 4 (“the symptom bothered or distressed me extremely). The DHSCL was chosen because it had been extensively used for the assessment of symptoms of depression across a wide variety of cultures including several East African populations (e.g. [54–58]). We applied the commonly used procedure of summing up the item-scores and dividing them by the number of items.

#### Impairment of functioning due to psychological problems

In order to gain more detailed knowledge about functional impairment in specific daily routines a local measure of functioning was developed, since other detailed instruments contain too many culture-bound questions. The process of developing the Luo Functioning Scale (LFS) followed an approach described by Bolton and Tang [59]. Fifty local informants were asked to list tasks that women and men must do regularly to care for themselves, for their family and for their community. The nine most frequently mentioned tasks (three per category) were compiled in separate functioning questionnaires for females and males. A tenth question about sexual interest/activity was added. Respondents were asked about the degree of difficulty they experienced in completing the tasks or activities in the past month. Answers were rated on a 3-point scale ranging from 0 (“no difficulties”) to 3 (“often can’t do task”). The causes of difficulties were documented for each item rated at least 1, and causes not referring to alcohol consumption or other psychological problems (e.g. lack of financial means) were not counted. An overall scale score was achieved by dividing the sum score by the number of validly answered items. The LFS has been successfully used in Northern Uganda before and was only slightly adapted in the scaling for the present research [52, 55].

#### Alcohol-related stigmatization

The Perceived Stigmatization Questionnaire (PSQ [60]) was shortened to an 11-item version representing the 2 factors “confused, staring and hostile behavior” (e.g. “People act surprised or startled when they see me.”, “People call me names.”) and “absence of friendly behavior” (e.g. “People treat me with respect.” [reverse coded]) in an earlier study [55]. Respondents’ answers concerning the frequency of stigmatizing behavior during the 4 weeks prior to the screening were coded on a 5-point scale ranging from 0 (“never”) to 4 (“always”). For each item the reason for discrimination was assessed as string variable. Only when participants gave a reason for stigmatization connected to their alcohol consumption the rating was considered for this study. An overall scale score was achieved by dividing the sum score by the number of validly answered items.

Apart from these, we assessed information that goes beyond the scope of the present article, namely history of abduction and displacement, history of violence in the family of origin, trauma exposure including war-related traumatic events, dependence-history and drinking motives (published elsewhere, [31]). Participants’ and their partners’ longitudinal information on experienced intimate partner violence, communication problems within the relationship, relationship quality and satisfaction, and parenting behavior can be found in the supplementary material. Where applicable we also interviewed a child between 8 and 13 years, for whom the participant was the guardian, before the *Treatment Camp* and at both follow-up time-points. Only few partners and children were available for interviews. Although their observations and experiences are invaluable, the statistical significance is limited due to the small sample size. Therefore, we report this data in the supplementary material.

### Data analyses

We conducted repeated-measures analyses of variance (ANOVAs) for all outcome measures with assessment time as within-subject factor. The assumptions of normality and sphericity were fulfilled if not otherwise specified. Reports on post-hoc comparisons are considerate of Bonferroni correction. Effect sizes (Hedges’s *g*) were computed for change from pre-assessment (PRE) to the 12 months follow-up (FU2). We used Hedges’s *g* rather than Cohen’s *d* since it is recommended for use with dependent measurements as is the case here. The built-in Bessel-correction reduces estimation bias especially with rather small sample sizes. Hedges’s *g* uses the average standard deviation as a standardizer [61]. Data analyses were carried out with SPSS Version 25.0 [62].

## Results

Table 2 summarizes the *Treatment Camp* participants’ development over time in terms of frequency and amount of substance use and symptom-based risk level according to the AUDIT. Reporting AUDIT scores below 8, at FU1 50% and at FU2 67% of participants classified as subthreshold consumers. Participants were not only drinking less frequently at FU1 and FU2, they also drank lower amounts on the occasions they were still drinking (Table 2). Out of the 25 participants 13 indicated smoking tobacco regularly pre-treatment. Twelve of them were available for follow-ups. They indicated a reduction of cigarette intake by 60% at FU2.

**Table 2:** Alcohol and Tobacco use over time

	PRE (n = 25)	FU1 (n = 24)	FU2 (n = 24)
Age tasting alcohol for the first time, mean (SD)	14.56 (6.12)		
Age when addiction was realized, mean (SD)	29.50 (10.90)		
Frequency of alcohol intake, N (%)			
never	–	5 (21)	3 (13)
monthly or less	–	7 (29)	9 (38)
2 to 4 times a month	–	1 (4)	6 (26)
2 to 3 times a week	6 (24)	7 (29)	4 (17)
4 or more times a week	19 (76)	4 (17)	2 (09)
Alcohol intake in standard drinks on a typical day with consumption, N (%)^a^			
1 or 2	–	8 (33)^b^	9 (38)^c^
3 or 4	–	2 (8)	5 (21)
5 or 6	–	3 (13)	4 (17)
7 to 9	3 (12)	4 (17)	3 (13)
10 or more	22 (88)	2 (8)	–
Classification according to risk level appropriate intervention, N (%)^d^			
Education (0–7)	–	12 (50)	16 (67)
Advice (8–15)	1 (4)	5 (21)	6 (25)
Advice, Counseling & Monitoring (16–19)	2 (8)	3 (13)	2 (8)
Specialist Diagnostics & Treatment (20–40)	22 (88)	4 (17)	–
Tobacco consumption in cigarettes on a typical day with consumption, mean (SD)	6.85 (4.19)^e^	2.21 (1.56)^f^	2.75 (1.06)^g^

*Note.* ^a^ One standard drink is defined as a drink containing 13 g of pure ethanol, e.g. 1 bottle of beer at 330 ml and 5%. ^b^ 5 abstinent participants. ^c^ 3 abstinent participants. ^d^ classifications follow the recommendations of the AUDIT manual, AUDIT scores in brackets. ^e^ *n* = 13 indicated smoking. ^f^ n = 12, two abstinent participants. ^g^ *n* = 12, no abstinent participants

### Effects of the *Treatment Camp* intervention on alcohol-related measures, depression and PTSD symptoms

A series of one-way repeated measures ANOVAs indicated that all alcohol-related outcomes differed significantly between time points (Table 3). For instance, there was a significant effect of time on the primary outcome, alcohol-related symptoms according to the AUDIT, *F* (2, 44) = 74.15, *p* < .001, η^2^ = .77. Pairwise comparisons of pre-treatment and follow-up scores revealed significant reductions on all AUD indicators (alcohol-related symptoms, drinking amount, drinking frequency, craving, obsession with alcohol and alcohol-related functioning impairment) from pre-assessment to 5-month follow-up. These reductions remained stable up to one year after treatment. There was no significant additional improvement between the two follow-up assessments. Effect sizes for time ranged without exception in the large range η^2^ = .32–.77 (Table 3). In addition to η^2^, we calculated Hedges’s *g* for change in symptomatology from pre-assessment to the 12-month follow-up (Table 3).

**Table 3:** Course of alcohol use, alcohol-related symptoms and consequences as well as comorbid psychopathology over time

	PRE (n = 25)	FU1 (n = 24)	FU2 (n = 24)	Statistic F-Value/ Χ^2^-Value	η^2 a^	Hedges’s *g*^b^
Alcohol-related symptoms (AUDIT), mean (SD)^c^	27.92 (7.33)	9.67 (8.92)	6.79 (3.83)	74.15***	0.77	3.49
Alcohol consumption in standard drinks on a typical day with consumption, mean (SD)^d^	23.19 (12.80)	4.72 (6.26)	2.90 (2.47)	48.19***^e^	0.69	2.13
Percentage of days with consumption, mean (SD)	69.76 (22.41)	18.13 (25.75)	19.59 (26.96)	45.02***^e, f^	0.68	1.96
Obsessive thoughts related to drinking (OCDS), mean (SD)^g^	12.00 (6.36)	4.83 (5.20)	5.09 (4.49)	18.76***^f^	0.47	1.21
Compulsive drinking behavior (OCDS), mean (SD)^g^	13.80 (4.52)	6.33 (6.16)	7.08 (4.58)	19.17***	0.47	1.43
Craving intensity in the past week, mean (SD)^h^	5.84 (2.70)	3.04 (3.21)	2.83 (2.71)	10.91***^f^	0.34	1.07
Craving frequency in the past week, mean (SD)^h^	5.36 (3.16)	2.46 (2.90)	2.61 (2.81)	10.39***^f^	0.33	0.89
Functioning impairment due to alcohol or other mental health related issues (LFS), mean (SD)^i^	0.31 (0.33)	0.06 (0.15)	0.08 (0.18)	10.27**^e^	0.32	0.84
Stigmatization because of alcohol-related problems (PSQ), mean (SD)^j^	0.82 (0.88)	0.26 (0.43)	0.25 (0.56)	9.86**^f, k^		0.75
PTSD Symptoms (PDS), mean (SD)^l^	2.08 (4.65)	1.39 (2.98)	1.88 (4.68)	0.05^k^		0.04
Depression Symptoms (DHSCL), mean (SD)^n^	2.13 (0.68)	1.47 (0.50)	1.70 (0.69)	9.49**^k^		0.61

*Note.* ^a^ Effect sizes of ≥ .01 are considered small, of ≥ .06 medium and of ≥ .14 large (*n* = 23). ^b^ Effect sizes are reported for PRE to FU2 only (n = 24). Hedges’s *g* is interpreted equivalent to Cohen’s *d*, i.e. values of ≥ .20 are considered small, of ≥ .50 medium and of ≥ .80 large. ^c^ possible score range: 0–40. ^d^ One standard drink is defined as a drink containing 13 g of pure ethanol, e.g. 1 bottle of beer at 330 ml and 5%. ^e^ The assumption of sphericity is violated, significance is reported according to the Greenhouse-Geisser correction. ^f^ reduced *n* = 22 for repeated measures ANOVA and *n* = 23 for Hedges’s *g*. ^g^ possible score range: 0–20. ^h^ possible score range: 0–10. ^i^ possible score range: 0–2. ^j^ possible score range: 0–4. ^k^ The assumption of normality is considerably violated, therefore significance is determined via the non-parametric Friedman test, Χ^2^-values are reported. ^l^ possible score range: 0–51. ^m^ possible score range: 1–4

significance: **p < .01, ***p < .001

Perceived stigmatization because of alcohol-related problems also reduced significantly over time. For this measure only the pairwise comparison of pre-treatment and one-year follow-up yielded a significant result, with a Hedges’s *g* coefficient in the medium range (.75). The initially low level of baseline PTSD symptoms did not change significantly over time, whereas comorbid symptoms of depression did. However, the significant reduction in symptoms of depression did not remain stable and had worn-off at the 12-month follow-up, with Hedges’s *g* = .61 still in the medium range.

**Fig. 3:**
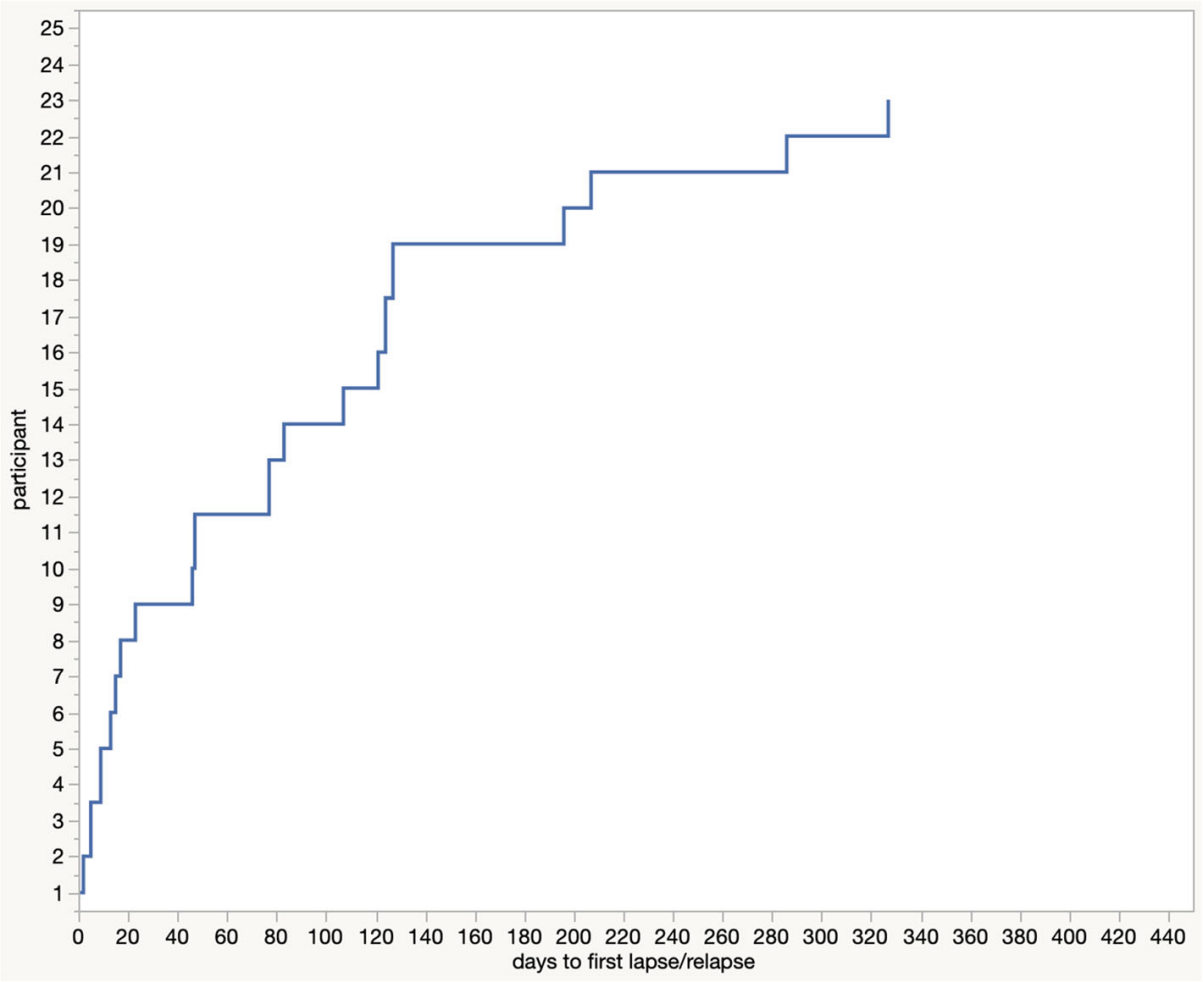
*Days to first lapse or relapse over the entire follow-up period. Note.* two participants did not lapse over the entire follow-up period

After the *Treatment Camp*, we assessed the number of days until the first lapse or relapse for each participant. After 23 days, 36% percent reported having drunk at least once. After 3 months, 56% had lapsed or relapsed and 76% after about 4 months. Up to the end of the entire assessment period, 92% had at least lapsed once (Fig. 3).

### Service user opinion, acceptability, feasibility, safety and costs

Apart from the participants’ quantitative data, qualitative interviews and data from partners and children documented that participants and their social environment predominantly reported positive change after the *Treatment Camp* (cf. Supplementary Tables 1 and 2, Additional File 1). The qualitative interviews showed that despite the use of medication participants could memorize the contents of the *Treatment Camp* well (cf. Supplementary Table 9, Additional File 2) and that they were generally convinced by the intervention elements. They identified many aspects as having been helpful to them during the *Treatment Camp* in their aim to quit drinking alcohol. Standard elements of detoxification and relapse-prevention programs were named, e.g. medication, nutrition supplements, being kept busy, psychoeducation on AUD and the negative effects of alcohol and learning about strategies to control the urge to drink. Additionally, participants also stressed the positive impact of feeling social connection and support through the group experience, and sharing testimonials. The accepting atmosphere created by the counselors was mentioned as well as supportive effects of the everyday morning meditation and prayer. The participants emphasized that developing insight that change is needed and noticing positive change in themselves already in the *Treatment Camp* helped them to stay on the right track. First positive reactions from their social environment outside the camp were also reported as having been helpful (for more details cf. Supplementary Table 3, Additional File 2). Fifty-two percent of the participants answered that they found nothing at all was not ideal or not helpful in the *Treatment Camp*. The other half openly shared aspects they considered not ideal, or which they perceived as a hinderance rather than a help. Difficulties in understanding the psychoeducation on negative effects of alcohol were mentioned, which participants partly ascribed to the side-effects of medication, partly to the way of teaching. Some found the counselors themselves not convincing, e.g. they considered some counselors to be under-qualified, or lacking experience, or to be unconvincing role-models. Some participants felt discouraged by misinformation, e.g. some felt the promised effect of medication did not set in. An offending communication style or attitude towards participants was also mentioned and predominantly attributed to one counselor. Some participants were distracted by bodily concerns, like pain or persistent craving despite medication. Others were irritated by fellow participants because they did not stick to the *Treatment Camp* rules. Internal barriers that prevented fully engaging with treatment were also mentioned, like inner resistance, or missing friends. Some participants thought the duration of the *Treatment Camp* was too short. Others wished for additional components, like physical therapy or games and exercises. A last aspect mentioned as not helpful during the Camp time was that their external social environment was not supportive, but rather mocking participants for being in the *Treatment Camp* (for all details cf. Supplementary Table 5, Additional File 2). For details on what aspects were mentioned as helpful or not helpful, respectively after the *Treatment Camp* phase cf. Supplementary Tables 4 and 6, Additional File 2. The fact that nobody dropped out of the *Treatment Camp* and only 6 participants did not join any of the voluntary meeting options after the *Treatment Camp* additionally emphasizes the high acceptance of the program by the participants.

Recruitment via radio calls and word of mouth communication worked well in this setting. It resulted in expressions of interest from individuals matching the program’s target group. Only 4 treatment seeking participants were not eligible since they were presenting with consumption on a lower level than the *Treatment Camp* was aiming at, and only 2 persons changed their mind concerning participation after having learned more about the intervention. Participants reported no problems in organizing the one-week inpatient stay. On the contrary, as already mentioned, many whished for a longer duration.

No adverse events (death, attempted suicide, unplanned hospitalization) occurred during the *Treatment Camp*. Nobody worsened concerning AUDIT-scores and drinking amounts. Two participants reported a meaningful increase in compulsive behaviors toward alcohol (OCDS subscale). Another participant reported meaningfully increased craving intensity and frequency on the visual analogue scales. Concerning symptoms of depression (DHSCL), two, and symptoms of PTSD (PDS), one participant reported meaningfully increased scores. No other medication than those indicated and planned as integral part of the detoxification were prescribed. All participants received diazepam that was gradually reduced to zero during the *Treatment Camp*. One participant was prescribed amitriptyline against depression symptoms. Two received carbamazepine to protect against withdrawal seizures and the medication was discontinued as soon as GRRH staff found it medically indicated. One of the latter two was later prescribed fluoxetine against depression symptoms. As expected, the development of some participants during the detoxification phase confirmed that psychiatric attention is indispensable for AUD-treatment in this context. Hallucinations were reported by ten participants during the daily monitoring consultations and were treated with haloperidol and promethazine by GRRH staff.

Costs per participant for the one week *Treatment Camp* were 390.000.- UGX (around 111 USD) of which 50.000 UGX were contributed by participants. Costs are inclusive of facilitators’ allowances, medication, venue, stationary and material used in the sessions, accommodation, catering, communication and the initial mobilization via radio.

## Discussion

This exploratory study evaluated a locally designed and implemented one-week community-based inpatient detoxification treatment complemented by psychosocial interventions aiming at psychoeducation and relapse prevention (cf. Figure 2). The approach is not primarily new concerning its content, but innovative and groundbreaking concerning its mode of service delivery and recruitment, which is highly adapted to the context of LMIC. Firstly, the mobile camp setup is ideally overcoming the lack of secondary and tertiary health facilities by covering large underserved areas consecutively. Secondly, it enables clients, who would neither have the means to travel nor to stay in a specialized rehabilitation center to use the service. Thirdly, the *Treatment Camp* is set up in a way to optimally imitate an inpatient setting, where patients can concentrate on recovery without distraction. Fourthly, the inpatient nature of the *Treatment Camp* overcomes many of the exclusion criteria that apply to home-detoxification programs or brief interventions that are the common alternative models of alcohol-related service delivery in LMIC [11, 14, 34, 35]. The combination of the inpatient setting with medical staff overseeing the provision of medication and dealing with physical concerns on the one hand and counselors or lay therapists providing sessions on psychoeducation and relapse prevention on the other hand helps to treat and monitor AUD patients intensively. This allows for the inclusion of individuals with severe AUD, with withdrawal symptoms, and comorbid psychiatric disorders. Lastly, since the program was designed and implemented in collaboration between the local NGO PACTA, local Caritas counselors, and staff of the local regional referral hospital, the risk of choosing culturally or contextually inappropriate or incomprehensible content or interventions was limited.

The recruitment strategy chosen by PACTA is well adapted to the requirements of resource-poor settings, where mobile phone- and TV-based, printed or internet-based information is not yet reaching the majority of people. Radio calls in combination with verbal transmission of information by community leaders eventually lead to word-of-mouth communication among the population, finally reaching most of the target-individuals. A positive side effect of announcing the *Treatment Camp* is that individuals and their families, who would not have known where to turn with an alcohol-related problem, learn about treatment options. This recruitment strategy might be more efficient than recruiting at public or private clinics as Nadkarni et al. [35] did. Primary care facilities as entry point seem pragmatic, yet Nadkarni et al. [35] reported a low consent rate of 23% next to a high dropout rate, which was not the case for the *Treatment Camp* approach with a consent rate of 89% and no dropouts during the *Treatment Camp* intervention. The low dropout-rate during treatment in the present study is exceptional for this patient-group [63].

Acceptability, feasibility and safety of the *Treatment Camp* approach was good. This is confirmed by quantitative and qualitative data from participants and their relatives. Recruitment reliably reached the targeted population, no participant discontinued the one-week program and no adverse events or emergency referrals occurred. In contrast, the pilot study by Nadkarni et al. [35] exploring the acceptability of a home-based detoxification and relapse prevention program in India lost 3 out of 11 (27%) participants in the detoxification-phase and 20 out of 27 (74%), who had joined the relapse component only. This vast contrast in dropouts might be partly explained by the differing recruitment strategies of the two exploratory studies. Moreover, PACTA’s *Treatment Camp* approach is likely to be safer than other (lay-)counselor-delivered services commonly implemented in LMIC, since complications in the detoxification phase can be directly and professionally handled. In fact, most publications about alcohol-related interventions in LMIC either don’t report on their dealing with withdrawal symptoms or exclude persons that are likely to develop withdrawal symptoms [11, 14].

However, health service delivery by NGOs or community-based organizations (CBOs) like the *Treatment Camp* described here, has one major disadvantage: its uncertain sustainability. Usually local nongovernmental service providers rely on funding from private donors or funds from bigger international agencies and development aid organization. Resources are granted for limited implementation periods and funding priorities are changing quickly, often resulting in ad hoc developed programs that can neither be thoroughly evaluated nor implemented permanently. A way forward would be the funding of scientific evaluation and sustainable implementation of verified developments by NGOs. In a subsequent step these could be considered for dissemination to public health service providers. In the case of the *Treatment Camp* approach eight further Camps followed in each of the eight districts of the Acholi sub-region. Recruitment, management, content and duration of these *Treatment Camps* followed the model of the one described here, including the participation of a Senior Psychiatric Clinical Officer and a Laboratory Technician from GRRH. Each time PACTA linked up with the main hospitals of the respective region for potential emergency referral.

This case series following *Treatment Camp* participants and their families up to one year after treatment found that 67% of the former AUD patients were either abstinent or considered subthreshold consumers at the last follow-up. Those who were drinking and smoking at the same time additionally reduced their cigarette intake by 60%. Alcohol-related symptoms, drinking frequency and amount (measured according to the AUDIT and TLFB) and indicators of craving (OCDS), including craving frequency and intensity, reduced significantly up to one year after treatment with large effect sizes (Hedges’s *g*) ranging from 0.89 to 3.49. Nadkarni et al. [35] found a significant difference between baseline and 3 months post recruitment assessments in daily alcohol consumption and heavy drinking for those participants who received home detoxification and relapse prevention counseling, but not for those who received relapse prevention counseling only. The first finding is in line with the current study that also combined both approaches and found high effect sizes on all alcohol-related measures. A single-arm trial with 185 Italian dependent drinkers, who attended a one-week inpatient detoxification program with a fixed-schedule drug regimen and accompanying non-pharmacological interventions was very similar to the present trial in treatment content and design and reported extremely similar AUDIT trajectories from pre-assessment to the 6- and 12-month follow-ups [64]. In Zambia, Sheik et al.’s [8] 7–10 days detoxification treatment combined with a 20-min relapse prevention intervention from the WHO mental health general action plan included the AUD patient and a relative as co-therapist, who was asked to help the patient to remain abstinent, join mutual-help groups and request further appointments in case of relapse. It was similar to the present program concerning its success, but differed concerning the location of service delivery, the type of aftercare, the applied outcome measures and the follow-up period. The treatment was carried out in the only psychiatric hospital of the country (Zambia), i.e. access to care for AUD patients, especially from the periphery of the country was likely to be limited and follow-up assessments were carried out up to 2 months only as opposed to 12 months in the present study.

We did not find any significant additional gain for the 19 participants that attended at least one session of the guided peer support groups (either AA meetings, or Caritas or GRRH open-topic psychosocial groups). The trajectory of first-time lapses after intervention followed results reported by Witkiewitz and Masyn [65], with a decreasing risk of lapsing over time. All except two participants lapsed at least once during the follow-up period, but still the reduction in drinking frequency, amount and related symptoms was significant and remained stable for more than one year post intervention. This is again in line with Witkiewitz and Masyn [65], who describe common patterns of post-lapse drinking in a large sample of American participants of a community-based alcohol treatment. They report the vast majority returns to abstinence or infrequent drinking following initial lapse and only few individuals show frequent heavy drinking after lapsing initially. Participants were openly sharing reasons for (re)lapsing in the qualitative interviews (cf. Supplementary Table 7, Additional File 2). Internal (physical or emotional) and external conditions triggering the urge to drink were mentioned prominently, e.g. the wish to overcome negative emotions, boredom, physical pain, or the wish to forget problems or being unable to withstand the former drinking environment. Other reasons were social pressure, e.g. from family, friends and during occasions where drinking is commonly expected.

Our measure of daily impairment in functioning was not restricted to AUD-related difficulties only, but included functioning impairment due to other psychopathologies as well. Self-reports of *Treatment Camp* participants indicated a significant improvement in functioning that was documented by a large effect size (Hedges’s *g* = 0.84). Perceived stigmatization because of alcohol consumption reduced significantly as well, with a slightly lower, medium effect size (Hedges’s *g* = 0.75). This might hint at a change in people’s attitude and behavior towards the former dependent drinkers and could be a sign of regained social status, possibly leading to a re-integration into productive work and community life. Qualitative statements of *Treatment Camp* participants back this interpretation (cf. Supplementary Tables 3, 4 and 8, Additional File 2). However, we can’t fully rule out that for some participants only their perception changed and not the actual attitude and behavior of their social environment. We observed a significant reduction in depression symptoms in the medium range (Hedges’s *g* = 0.61), yet not for PTSD symptoms. A similar decrease in depression symptoms was reported by Oliva et al. [64] in their sample of Italian AUD patients attending a one-week inpatient detoxification program. Depression symptoms may have reduced as a function of participants’ reduced alcohol use, although we can’t prove this assumption, since we lacked the power for mediation analyses. The lack of change in symptoms of PTSD is not surprising, since no PTSD-specific treatment components were implemented during the *Treatment Camp*. Moreover, only few PTSD symptoms were reported at pre-assessment.

Concerning the trajectories of communication problems within their relationship, relationship satisfaction and quality, the male participants generally report less change than their partners (cf. Supplementary Table 1, Additional File 1). This result is plausible, since the male participants were the ones who were in treatment and possibly changed their behavior within the relationship parallel to their drinking habits. Interestingly large changes in IPV were reported by both partners. This implies that physical and emotional violence reciprocally reduced once the male partner had changed drinking habits. This finding is contrary to what was recently reported by Tol et al. [36], who found that the reviewed alcohol-focused intervention studies did not show benefits on IPV. In line with the couple-results, the participating guardians and their children report a considerable decrease of harsh parenting and family violence including neglect (cf. Supplementary Table 2, Additional File 1). Interestingly, both parties agree on the decline of physical violence, but not on emotional/psychological maltreatment. Children reported an improvement on emotional maltreatment, whereas the participating guardians reported not having changed their psychological aggression.

In addition to the above mentioned strengths of the *Treatment Camp* approach itself, several strengths of the evaluation study stress its uniqueness. This is the first study ever examining the feasibility, acceptability and safety of a mobile, community-based inpatient treatment model for AUD and one of the few dealing with severe AUD in LMIC. The evaluation of this innovative service delivery approach was carried out by an organization different from those who designed and implemented the interventions. This constellation is likely to lead to less bias in the data, since assessors are not feeling a strong commitment towards the program and participants are less likely to answer in socially desirable ways. At each follow-up participants were interviewed by assessors they had not interacted with before. We had hardly any dropouts, with only one participant lost at each follow-up assessment. This is rare in this clientage, especially considering the long follow-up period [64]. The latter is a strength in itself, since most alcohol-related intervention evaluations commonly use shorter follow up periods of 3 to 6 months [11, 13, 14]. This study used a mixed method approach and complemented quantitative data with qualitative data, which has not been done for withdrawal treatments in this context before. Qualitative data is especially valuable in this early stage of intervention evaluation in order to learn which and how much content dependent drinkers can grasp during the detoxification phase. The feedback of participants on helpful and not helpful aspects of the *Treatment Camp* and the time after will facilitate adjustments to this emerging intervention model and contains valuable information for other intervention endeavors as well (cf. Additional File 2). This study is meeting the call for more service user involvement in LMIC on two different levels [66]. Firstly, PACTA staff includes former AUD patients who integrate their experiences and know-how from management to intervention planning and implementation. Secondly, *Treatment Camp* participants were asked for their feedback on treatment content and processes.

Although their number is small, we included participants’ partners and children and can at least descriptively report their data in the Additional File 1. This longitudinal data from involved family members complements and partly validates the reports of *Treatment Camp* participants. In contrast to other studies in the field (e.g. Nadkarni et al. [35]) we did not solely rely on the AUDIT for the assessment of AUD, but pre-assessments were confirmed by GRRH staff at entry into the *Treatment Camp*. Moreover, we did not only assess alcohol-related measures, but also perceived stigmatization, functioning impairment and other pathologies.

This study also has several limitations that should be noted. Since the organizations involved in implementing the *Treatment Camp* did not design its realization as a comparative trial, the absence of control arms means that participants’ changes could also be due to spontaneous remission or to a placebo effect of an actually inefficacious intervention that could have also spread to the non-participating family members. We consider spontaneous remission on a large scale unlikely, since participants reported to have realized addiction-related problems at around 29.5 (SD = 10.90) years of age, yet they were on average 40.6 (SD = 10.71) years old at entry into the *Treatment Camp*. This implies rather long histories of addiction. Participants were recruited following a radio-call, i.e. they represented a self-selected population which limits generalizability. Participants were almost exclusively males, which limits generalizability to females with AUD. However, this imbalance was to be expected and is reflecting the prevalence of AUD in the given context. AUD is still almost exclusively a male problem in Uganda [5, 6, 8, 19]. Although the information provided by partners and children validated the participants’ information to a certain extend our findings are relying on self-reports that could have been biased. Social desirability and feelings of shame might have played a role during follow-up assessments, however, studies suggest that self-report on alcohol is quite accurate [67, 68]. The instruments for craving were implemented for the first time in the given context and still would have to be validated.

## Conclusion

This single-armed evaluation study is one of the few contributing to the scientific evaluation of treatment possibilities for AUD in LMIC, especially those targeting severe addiction. The community-based *Treatment Camp* approach provides initial evidence that in light of raising prevalence rates of AUD in LMIC [19] and extremely limited access to specialized care, innovative approaches can be promising ways of tailoring interventions to challenging settings, without losing effectiveness. Like another recently tested pragmatic solution, the home-detoxification and relapse prevention program piloted by Nadkarni et al. [35], the *Treatment Camp* approach also seems to be resource-efficient. Both approaches are superior concerning accessibility than having to resort to specialized health facilities in urban centers. The *Treatment Camp* approach has additional advantages over home-detoxification, since due to its inpatient-character it is by far less limited by exclusion criteria. Next steps should include extending these approaches in LMIC to test their efficacy and effectiveness in larger and thoroughly designed trials including active control groups before possibly scaling them up for routine use in diverse resource-poor settings. Assessment of family members to investigate potential changes in relationships and family violence should be integrated into these research endeavors.

## Supplementary Information

**Additional File 1. ***Partners’ and children’s reports of relationship quality and family violence* is providing information on assessment instruments and results of the pre-, and follow-up data provided by the participants’ partners and children.

**Additional File 2. ***Results of the qualitative interviews on the Treatment Camp and the rehabilitation process afterwards* is providing information on the procedures and results of the qualitative analysis concerning the research questions: a) What elements of the *Treatment Camp* were perceived as helpful / not helpful by the participants concerning their aim to quit drinking, b) What elements were perceived as helpful / not helpful after the inpatient phase and c) What factors were perceived as causing a relapse into drinking or successful abstinence or harmless intake respectively.

## Data Availability

The datasets used and/or analyzed during the current study are available from the corresponding author on reasonable request.

## Abbreviations

LMIC: Low and middle income country/countries
AUD: Alcohol Use Disorder
IPV: Intimate Partner Violence
PTSD: Posttraumatic Stress Disorder
AUDIT: Alcohol Use Disorders Identification Test
TLFB: Timeline Follow Back Interview
OCDS: Obsessive Compulsive Drinking Scale
PDS: Posttraumatic stress Diagnostic Scale
DHSCL: Depression-section of the Hopkins Symptom Checklist
PSQ: Perceived Stigmatization Questionnaire
PRE: pre-assessment before the *Treatment Camp*
FU1/FU2: follow-up 1 (5 months after the *Treatment Camp*)/follow-up 2 (12 months after the *Treatment Camp*)
NGO: Nongovernmental Organization
CBO: Community-based Organization
PACTA: Program for Prevention, Awareness, Counseling and Treatment of Alcoholism
GRRH: Gulu Regional Referral Hospital
UGX: Uganda Shilling
USD: US Dollar
