# Supplementary material for: Treating alcohol use disorder in the absence of specialized services – evaluation of the moving inpatient Treatment Camp approach in Uganda
Source: BMC Psychiatry. 2021 Dec 1;21:601. doi: 10.1186/s12888-021-03593-5 (PMC8638348; doi:10.1186/s12888-021-03593-5)
Supplement: Supplementary file 1 — Additional File 1. Partners’ and children’s reports of relationship quality and family violence is providing information on assessment instruments and results of the pre-, and follow-up data provided by the participants’ partners and children. [file 12888_2021_3593_MOESM1_ESM.pdf]

Supplement 1: Partners' and children's reports of relationship quality and family violence

Methods

Of the 25 allocated individuals, nine male participants indicated potential participation of their intimate partners. One woman was not available for the follow-up assessments and one at FU1 and FU2 only. Eight of the participants' children qualifying for the eligible age-range 8 to 13 and living with the participant agreed to take part. Two of the children were not available for the FU2 assessment.

Measures

Relationship Quality

Relationship communication was assessed using Gottman's the Four Horsemen of the Apocalypse Questionnaire, reflecting the concept of four communication styles that predict relationship failure: a) criticism, defined as verbally attacking the partner's personality or character (e.g: "I feel attacked or criticized when we talk about our disagreements), b) contempt, defined as attacking the partner's sense of self with an intention to insult or psychologically abuse her/him (e.g.: "I feel basically disrespected"), c) defensiveness, defined as seeing oneself as the victim in efforts to ward off a perceived attack and reverse the blame (e.g.: "I am just not guilty of many of the things I get accused of") and d) stonewalling, defined as withdrawing from the relationship as a way to avoid conflict in efforts to convey disapproval, distance and separation (e.g. "I sometimes just calm up and become quiet") (Gottman et al., 1998). The scale consists of 33 items that were designed in a dichotomous answer format (true/false). Although the scale has been used in various studies and its reliability and validity has been claimed (Cornelius & Alessi, 2007; Gottman, 2011), there are no consistent instructions on how to sort items to the four dimensions (Lute, 2015). Since researchers having used rater-consensus and factor analytic approaches don't agree on item allocation, we decided to simply use one score representing communication problems in general. We calculated the mean of all 33 items, i.e., scores between > 0.5 and 1.0 indicate the majority of communication problems have been rated as true.

General relationship satisfaction was assessed using the Relationship Assessment Scale (RAS; Hendrick, 1988). The measure consists of 7 items scored using five-point Likert-type scales, with 2 items being reverse coded. It was designed to be a brief, easily administered and scored measure of overall satisfaction in romantic relationships. Items are globally formulated, e.g. "How well does your partner meet your needs?" or "How good is your relationship compared to most?". A mean score is calculated using all items, i.e. scores below 2 indicate dissatisfaction, since the anchor 2 is defined as average. Research has provided evidence that the RAS has good reliability and concurrent validity with a number of the subscales of the Love Attitudes Scale and Dyadic Adjustment Scale (Hendrick, 1988).

The Dyadic Adjustment Scale (DAS; Spanier, 1976) is a popular self-report measure of the quality of intimate relationships consisting of four subscales: dyadic consensus (13 items; the degree to which the couple agrees on matters of importance to the relationship), dyadic satisfaction (10 items; the degree to which the couple is satisfied with their relationship), dyadic cohesion (5 items; the degree of closeness and shared activities experienced by the couple), and affectional expression (4 items; the degree of demonstrations of affection and sexual

relationships). Whereas the original version consists of 32 items rated on five- to seven-point Likert-type scales or dichotomous items, we created a short version on the basis of the first 15 items only. We decided to shorten the questionnaire to avoid changing scale anchors that would have easily confused respondents, to keep the interview length manageable and to avoid repeating questions already asked in the RAS. Additionally, we removed 5 items that were considered inappropriate for the given context after discussions with the experienced bilingual mental health counselors that supported us in instrument translation and adaptation. These were items asking about career decisions and leisure time interests. The remaining 10 items reflected two of the original 4 constructs, dyadic consensus (8 items) and affectional expression (2 items). The 10 items were scored on a six-point Likert-type scale. We calculated the mean using all items, i.e. scores above 2.5 indicated that participants predominantly agreed and were satisfied with how their partners were handling important issues, pointing towards more positive dyadic adjustment.

Family Violence

In order to assess past-year prevalence and level of partner violence experienced by the female and male partners we adapted the Composite Abuse Scale (CAS; Hegarty, 2007). The original scale consists of 30 items forming the sub-scales emotional abuse, physical abuse, severe combined abuse including sexual violence, and harassment (Hegarty et al., 1999). The frequency of abusive acts is coded on a six-point Likert-type scale ranging from 0 (never) to 5 (daily). We kept this scaling, but made gender- and context-specific adaptations. The subscale of harassment (4 items) and one item of sexual violence had already been omitted in an earlier version of the questionnaire that had been used with female respondents exclusively because items appeared to be contextually inappropriate (Saile et al., 2013). For this research we created a male version for the first time and introduced two new items developed together with the bilingual mental health counselors that supported us in instrument translation and adaptation. They had extensive experience with counseling in the communities and knew from their male and female clients about possible acts of intimate partner violence. The two items asking about sexual violence within the relationship ("tried to rape me" and "raped me") were omitted in the male version. Instead the two newly developed items of emotional/psychological abuse were introduced in the male version of the questionnaire. Additionally, the wording of three items had to be adapted in the male version, without changing the assessed concept. A previous version for exclusively women had been used in Northern Ugandan already and demonstrated good psychometric properties (Saile et al., 2013). Factor analysis suggested four factors: isolation (7 items), emotional/psychological abuse (8 items for women, 10 items for men), sexual abuse (2 items, women only), and physical abuse (8 items). We only report the overall level of intimate partner violence here, summing up the frequency codings for acts of intimate partner violence in the past year.

We used the Parent-Child Conflict Tactics Scales (CTSPC; Straus et al., 1998) to assess the participants' self-reported acts of psychological and physical aggression in difficult situations towards the participating child. Eighteen items differentiate between psychological aggression, corporal punishment, physical abuse and severe physical abuse. Three items additionally assess neglect. Four items code nonviolent discipline methods. The original scale uses an eight-point Likert-type scale to measure lifetime and one-year prevalence as well as frequency of specific acts given the act occurred at least once. In the current study, we restricted

the coding to a dichotomous answer format asking whether acts happened “ever in life” and whether they happened “in the past month”. The CTSPC has been adapted and used in the described format in studies conducted in Northern Uganda before (Saile et al., 2014). We calculated the cumulative number of different types of aggressive parenting behaviors and neglect excluding items on non-violent discipline.

Aversive events experienced or witnessed at home by the participating child were assessed using a 41-item event-checklist of family violence (FV). Events were answered in a dichotomous format as present “ever in life” and “in the past month”. Presented here are the events answered positively for the past month. They were summed up in total and per subcategory: physical abuse including neglect (13 items), emotional abuse including neglect (17 items), sexual abuse (4 items) and witnessed violence at home (7 items). The questionnaire included items taken from two standard checklists for traumatic experiences in childhood, the Early Trauma Inventory (Bremner et al., 2000) and the Childhood Trauma Questionnaire (Bernstein et al., 1994). The scale is conceptually close to the parent-report via the CTSPC with the main difference that children did not report on abusive acts from a referent guardian, but from any family member who was possibly involved in education and disciplining the child. A shorter version of the questionnaire has been used in Northern Uganda (Olema et al., 2014; Saile et al., 2016) and in other cross-cultural contexts (Catani et al., 2008).

Supplementary Table 1

Course of relationship communication, satisfaction and quality and intimate partner violence as indicated by both partners

	PRE	FU1	FU2	Hedges’s g^a
**communication problems** (The four Horsemen Questionnaire), mean (SD)[b]				
male partner (n=9)	0.52 (0.29)	0.34 (0.29)	0.39 (0.32)[c]	0.38
female partner (n=8)	0.68 (0.32)	0.55 (0.26)	0.52 (0.29)	0.47
**relationship satisfaction** (RAS), mean (SD)[d]				
male partner (n=9)	2.68 (0.72)	2.95 (0.68)	2.88 (0.86)[e]	0.22
female partner (n=8)	1.59 (0.89)	2.20 (0.66)	2.04 (1.07)	0.41
**relationship quality** (DAS), mean (SD)[f]				
male partner (n=9)	4.07 (0.74)	4.17 (0.70)	3.94 (1.08)[e]	-0.12
female partner (n=8)	1.95 (1.33)	2.89 (1.35)	2.95 (1.39)	0.65
**intimate partner violence in the past year** (CAS), mean (SD)[g]				
male partner (n=9)	20.56 (18.12)	8.89 (13.33)	1.71 (2.75)[c]	1.26
female partner (n=8)	18.80 (13.22)	15.41 (20.91)	6.58 (11.80)	0.87

Note. [a] Effect sizes are reported for PRE to FU2 only. Hedges’s g is interpreted equivalent to Cohens d, i.e. values of ≥ .20 are considered small, of ≥ .50 medium and of ≥ .80 large. [b] possible

score range: 0-1. [c] n = 7. [d] possible score range: 0-4. [e] n = 8. [f] possible score range: 0-5. [g] possible score range: 0-125.

Supplementary Table 2

Course of family violence as indicated by the AUD-affected parent (CTSPC) and one child between 8-13 living in the same household (FV)

	PRE	FU1	FU2	Hedges's g[a]
**parent report on harsh parenting in the past month** (CTSPC), mean (SD)[b] (n=8)	2.88 (1.55)	2.75 (2.55)	1.71 (1.50)[c]	0.67
corporal punishment[d]	0.5 (0.53)	0.63 (0.74)	0.29 (0.49)[c]	0.36
physical maltreatment[e]	0.5 (0.76)	0.13 (0.35)	0.14 (0.38)[c]	0.52
severe physical maltreatment[f]	0.25 (0.46)	0 (0)	0 (0)[c]	0.67
psychological aggression[g]	1 (0.76)	1.5 (1.41)	1.14 (0.90)[c]	-0.15
neglect[h]	0.63 (0.52)	0.50 (0.76)	0.14 (0.38)[c]	0.94
**child report on family violence in the past month** (FV), mean (SD)[i] (n=8)	5.13 (3.52)	4.25 (4.06)	0.71 (1.11)[c]	1.47
FV experienced physical[j]	2.13 (1.25)	1.13 (0.99)	0.29 (0.49)[c]	1.69
FV experienced sexual[k]	0 (0)	0 (0)	0 (0)[c]	-
FV experienced emotional[l]	2.50 (2.39)	2.63 (3.38)	0.29 (0.76)[c]	1.08
FV witnessed[m]	0.50 (0.53)	0.50 (0.93)	0.14 (0.38)[c]	0.68

Note. [a] Effect sizes are reported for PRE to FU2 only. Hedges's *g* is interpreted equivalent to Cohens d, i.e. values of ≥ .20 are considered small, of ≥ .50 medium and of ≥ .80 large. [b] possible score range: 0-21. [c] n = 7. [d] possible score range: 0-4. [e] possible score range: 0-5. [f] possible score range: 0-4. [g] possible score range: 0-5. [h] possible score range: 0-3. [i] possible score range: 0-41. [j] possible score range: 0-13. [k] possible score range: 0-4. [l] possible score range: 0-17. [m] possible score range: 0-7.

References

Bernstein, D. P., Fink, L., Handelsman, L., Foote, J., Lovejoy, M., Wenzel, K., Sapareto, E., & Ruggiero, J. (1994). Initial Reliability and Validity of a New Retrospective Measure of Child-Abuse and Neglect. *American Journal of Psychiatry*, *151*(8), 1132–1136. https://doi.org/10.1176/ajp.151.8.1132

Bremner, J. D., Vermetten, E., & Mazure, C. M. (2000). Development and preliminary psychometric properties of an instrument for the measurement of childhood trauma: The early trauma inventory. *Depression and Anxiety*, *12*(1), 1–12. https://doi.org/10.1002/1520-6394(2000)12:1<1::AID-DA1>3.0.CO;2-W

Catani, C., Schauer, E., & Neuner, F. (2008). Beyond individual war trauma: Domestic violence against children in Afghanistan and Sri Lanka. *Journal of Marital and Family Therapy*, *34*, 165–176. https://doi.org/10.1111/j.1752-0606.2008.00062.x

Cornelius, T. L., & Alessi, G. (2007). Behavioral and Physiological Components of Communication Training: Does the Topic Affect Outcome? *Journal of Marriage and*

Family, *69*(3), 608–620. https://doi.org/10.1111/j.1741-3737.2007.00395.x

Gottman, J. M. (2011). *Reliability and Validity of the Sound Relationship House Scales*. https://www.johngottman.net/wp-content/uploads/2011/05/Reliability-Validity-of-the-Sound-Relationship-House-Scales.pdf

Gottman, J. M., Coan, J., Carrere, S., & Swanson, C. (1998). Predicting Marital Happiness and Stability from Newlywed Interactions. *Journal of Marriage and the Family*, *60*(1), 5. https://doi.org/10.2307/353438

Hegarty, K. (2007). *Composite Abuse Scale Manual*.

Hegarty, Kelsey, Sheehan, M., & Schonfeld, C. (1999). A multidimensional definition of partner abuse. *Journal of Family Violence*, *14*(4), 399–415. https://doi.org/10.1023/A:1022834215681

Hendrick, S. S. (1988). A Generic Measure of Relationship Satisfaction. *Journal of Marriage and the Family*, *50*(1), 93. https://doi.org/10.2307/352430

Lute, M. (2015). The relationship between Gottman's Four Horsemen of the Apocalypse, mindfulness, and relationship satisfaction. In *Theses and Dissertations*. Indiana University of Pennsylvania.

Olema, D. K., Catani, C., Ertl, V., Saile, R., & Neuner, F. (2014). The Hidden Effects of Child Maltreatment in a War Region: Correlates of Psychopathology in Two Generations Living in Northern Uganda. *Journal of Traumatic Stress*, *27*(1), 35–41. https://doi.org/10.1002/jts.21892

Saile, R., Ertl, V., Neuner, F., & Catani, C. (2014). Does war contribute to family violence against children? Findings from a two-generational multi-informant study in Northern Uganda. *Child Abuse & Neglect*, *38*(1), 135–146. https://doi.org/10.1016/j.chiabu.2013.10.007

Saile, R., Ertl, V., Neuner, F., & Catani, C. (2016). Children of the postwar years: A two-generational multilevel risk assessment of child psychopathology in northern Uganda. *Development and Psychopathology*, *28*(2), 607–620. https://doi.org/10.1017/S0954579415001066

Saile, R., Neuner, F., Ertl, V., & Catani, C. (2013). Prevalence and predictors of partner violence against women in the aftermath of war: A survey among couples in Northern Uganda. *Social Science & Medicine*, *86*, 17–25. https://doi.org/10.1016/j.socscimed.2013.02.046

Spanier, G. B. (1976). Measuring Dyadic Adjustment: New Scales for Assessing the Quality of Marriage and Similar Dyads. *Journal of Marriage and the Family*, *38*(1), 15. https://doi.org/10.2307/350547

Straus, M. A., Hamby, S. L., Finkelhor, D., Moore, D. W., & Runyan, D. (1998). Identification of child maltreatment with the Parent-Child Conflict Tactics Scales: development and psychometric data for a national sample of American parents. *Child Abuse Negl*, *22*(4), 249–270.
