# Supplementary material for: Treating alcohol use disorder in the absence of specialized services – evaluation of the moving inpatient Treatment Camp approach in Uganda
Source: BMC Psychiatry. 2021 Dec 1;21:601. doi: 10.1186/s12888-021-03593-5 (PMC8638348; doi:10.1186/s12888-021-03593-5)
Supplement: Supplementary file 2 — Additional File 2. Results of the qualitative interviews on the Treatment Camp and the rehabilitation process afterwards is providing information on the procedures and results of the qualitative analysis concerning the research questions: a) What elements of the Treatment Camp were perceived as helpful / not helpful by the participants concerning their aim to quit drinking, b) What elements were perceived as helpful / not helpful after the inpatient phase and c) What factors were perceived as causing a relapse into drinking or successful abstinence or harmless intake respectively. [file 12888_2021_3593_MOESM2_ESM.pdf]

Supplement 2: Results of the qualitative interviews on the Treatment Camp and the rehabilitation process afterwards

Methods

We implemented semi-structured interview questions to gain data on three themes: a) What elements of the Treatment Camp were perceived as helpful / not helpful by the participants concerning their aim to quit drinking, b) What elements were perceived as helpful / not helpful after the inpatient phase and c) What factors were perceived as causing a relapse into drinking or successful abstinence or harmless intake, respectively. Interviewers were trained to follow an interview guide with obligatory open-ended questions and were instructed to note down the interviewees' answers. The opening question was chosen to be broad to help building up rapport and dialogue and asked about how participants were generally doing since the Treatment Camp (at the 5-month-follow-up) or the last interview (at the 12-month-follow-up). Follow-up questions were whether they had noticed any changes in their behavior at home, in the relationship with their partner, in their role as a father/mother, employee, and neighbor or friend and what they associated potential changes with. The next question dealt with what contents of the Treatment Camp the respective participant still remembered to facilitate the questions mentioned under a) to c). Interviewers were instructed to get detailed examples and to follow-up statements by asking why the participants labeled these issues as helpful or not helpful, respectively. The questions on what Treatment Camp content participants still remembered and the questions depicted under a) were asked at the 5-month-follow-up only.

Our analytic procedure followed the framework method (Gale et al., 2013). Participants' answers were entered as continuous text per question into the data base. The analysis team read each statement of each participant thoroughly to get familiar with the entire data set. Notes could already be taken at this stage to not lose ideas stemming from first impressions. Throughout the process, each of the three team members was encouraged to note down ideas, impressions, irritations and possible interpretations in a separate file as analytic memos for later joint discussion. The three team-members started off with independently coding the answers of the same three participants (open coding). After each new unit of meaning a column was entered and the extracted content was described with a label or code of few words. In case the code or label was not self-explicatory, in a second column notes could be taken to re-visit the ideas, questions or explanations later. For identical content within the statements the same codes were used, for differing content new codes were developed until all statements of these first three participants were fully depicted in codes. After the three team-members had finalized open coding for the three participants, the labels and codes were discussed. In case of differences in wording and expressions, we checked whether the team-members identified the same content and meaning as important. If this was the case, the better term was agreed upon. If this was not the case, content and meaning were discussed and a consensus was reached. After discussion, we agreed on a set of codes and provided brief definitions. This resulted in the initial analytical framework, that was recorded in the emerging codebook. Two team-members then independently coded three more participants' statements using the initial analytical framework, taking care to note any new codes or labels as in the previous step for statements that did not fit the existing set of codes. All three team-members then met again to discuss additional codes and disagreements and a revised analytical framework was established incorporating new and refined codes. Conceptually closely related codes were grouped together. The last two steps

were repeated until all statements of the 25 Treatment Camp participants were marked with a code and no new codes had to be developed. A final analytic framework was established that consisted of codes, clustered into categories, each with a brief explanatory description of their meaning and especially representative word by word examples.

Supplementary Table 3

Elements and aspects that were indicated at the 5-month-follow-up as having been helpful in the Treatment Camp inpatient phase concerning the aim to quit drinking alcohol (code) clustered in broader helpful components (category)

Category	Code	Representative direct quotes
Basic detoxification components	Unavailability of alcohol	*"…I stopped drinking…"; "…no easy access to alcohol…"*
	Change of environment	*"…change of environment…"*
	Medication	*"…the medication have been helpful because it was removing the alcoholic content in my blood…"; "…the medication I received helped me…"*
	Nutritional supplements	*"…nutrition therapy…"*
	Healthy nutrition	*"…good diet (helped people a lot)…"*
	Being kept busy	*"…avoiding idleness…"*
Imparting of knowledge on alcohol, AUD, its negative consequences and strategies on overcoming AUD	Psychoeducation	*"…the advice was good and the teaching…"*
	Psychoeducation on negative effects of alcohol	*"…the counseling the facilitators were giving us on the dangers of alcohol…"*
	Psychoeducation on negative health effects of alcohol	*"…talking about the dangers of alcohol to our health it helps me a lot…"*
	Psychoeducation on negative social effects of alcohol	*"…also what was being taught e.g. how addicts fail to take care of his or her family…"*
	Psychoeducation on positive consequences of being sober	*"…we were taught that when you do not consume alcohol then you will become healthy…"*
	Psychoeducation via movies	*"…movies were helpful…"*
	Everything learned in the Treatment Camp	*"…what we learnt from there (Treatment Camp)…"*
	Psychoeducation on self-control strategies	*"…steps in reducing taking alcohol (amount)…"*
Interactive single and group exchange on the impact of alcohol on the lives of participants	Counseling	*"…being counseled by counselor…"*
	Testimonials	*"…personal testimonies e.g. beating wife…"; "…thinking back helped me because I saw the way I was punishing myself…"*

Undetermined social support	Knowing one is not alone with alcohol problems	*"…someone else has the same problem…"*
	Sharing with others	*"…the good ideas I was sharing with fellow participants…"; "…you have someone to talk to about problems…"*
	Social connection	*"…it has made me know many people and we have become friends…"*
Basic counseling attitude	Accepting atmosphere by facilitators	*"…atmosphere ([…]high acceptance by facilitators, no borders no matter whether highly educated or not)…"*
	Free interaction	*" atmosphere (free interaction without borders […])…"; "… chance to tell what happened…"*
Spirituality/relaxation	Meditation/Prayers	*"…the chances of meditation making us to think back with reference to bible…"*
Developing insight and awareness that a change in drinking behavior is needed	Seeing negative effects of drinking in fellow participants	*"…because seeing friends who were wasted by alcohol helped me to quit alcohol…"; "…some people looked sick by appearance…"*
	Awareness of health problems	*"…the testing has been helpful because it has made them know what problems I have in my body...“; "…my condition plus drinking did not match…"*
	Awareness of need for help	*"…realized he […] needed help…", "…I realized that my life is on danger..."*
	Influence on reconsidering consumption	*"…it has made me to think of stopping taking it..."; "…it has helped me in that I am able to reduce on the amount of alcohol…"*
Noticing positive change related to alcohol	Losing interest in alcohol	*"…I no longer value alcohol the way that I did before…“; "…I don't want to smell alcohol…."*
	No craving	*"… I no longer crave for alcohol…"*
Noticing positive change in oneself	Change in appetite	*"…because I have seen that if I leave alcohol I feed well…"; "…I can eat and I used not to have good appetite…"*
	Improved social skills	*"…It has given me skills to stay with people…"*
	Improved problem-solving skills	*"…changed my way of solving problems…"*
	Chance to practice skills	*"…gave him a chance to practice his leadership skills …"*
	Improved health	*"…improved health condition…"*
	Improved physical appearance	*"…changed bodies of people, wrinkles started to disappear from faces..."; "I became smart not like these other days…"*
	Regained ability to work	*"…and it has made me now able to do my garden works..."*
	Change in Life through Treatment Camp	*"…training gave constructive way of life"; "…changed my life from bad to good…"*

Positive reactions from social environment	Positive change in attitude towards participant	*"…people trust me at home…"*
	New job offers	*"…and people can offer me casual labor…"*
	Support from social environment	*"…friends gave me their support…"*
	Improved family life	*"…brought happiness to my […] family…"; "…improved relationship…"*

Supplementary Table 4

Elements and aspects that were indicated at the 5- and 12-month-follow-up as having been helpful after the Treatment Camp inpatient phase concerning the aim to quit drinking alcohol (code) clustered in broader helpful components (category)

Category	Code	Representative direct quotes
Noticing positive change related to alcohol	Reduced drinking	*"…I started drinking less…"; "…stay sober most times…"*
	No craving	*"…no more temptation to drink…"; "…not craving…"*
	Sobriety	*"…I stopped drinking…"*
Noticing positive change in oneself	Clear mind	*"…sober mind…"; "…think straight…"*
	Peace of mind	*"…I was able to have peace of mind…."*
	Improved health	*"…my health improved…"*
	Improved energy	*"…my energy increased…"*
	Improved nutrition	*"…I had gained 5 kilos from the camp and was eating well so alcohol could not overpower me..."*
	Improved appetite	*"…my appetite improved..."*
	Improved physical appearance	*"…smartness improved…"*
	Improved self-control	*"…you have more control to stay sober…"*
Intentional recall of content of the Treatment Camp	Content of Treatment Camp/PACTA training	*"…when I remember the teaching it helps me to be sober…"*
	Reflection on negative health effects of alcohol	*"…their teachings about health concerns…"*
	Reflection on negative effects of alcohol	*"…I thought to myself if I continue taking alcohol I will not be able to take care of my animals and digging…"; "…I decided to stop drinking after learning the dangers of drinking…"*
	Testimonials	*"…testimonials…"*
	Meditation/Prayers	*"…Serenity Prayer every day…"; "because when I pray to God he can change my attitude towards drinking…"*
Keeping one's mind set on sobriety	Active decision	*"…personal decision to quit drinking…"*
	Self-reflection	*"…educated me a lot to start realizing what I never used to…"; "…self-reflection to my life style…"*
	Following example of others/role models	*"…use experiences of other people to leave drinking…"; "…seeing changes from others…"*

	Realizing negative effects of alcohol on other drinkers	*"…other drunkards look bad so she doesn't want to be like them anymore…"*
	Further reading on addiction	*"…reading liter alive on addiction – newspaper…"*
	Feeling obligation towards supporters	*"…I did not want to betray the people who offered us the alcohol treatment and also not to disappoint those who advice me positively…"*
Stimulus control strategies	Avoiding drinking friends	*"…staying away from friends who drink a lot…"*
	New social environment	*"…new group of friends…"*
	Avoiding drinking places	*"…decision to avoid drinking places…"*
	Staying at home	*"…stayed at home doing house hold work…"*
	Change in Lifestyle	*"…changed my way of life…"*
	Change in work routine	*"…changed my work routine…"*
Distraction strategies	Being busy (work, hobbies, sports)	*"…books to read…"; "…then he engages himself in active things such as riding bike instead of drinking…"; "…staying busy with handcraft – that took most of my time…"*
Elements of professional aftercare	Caritas counseling	*"…Caritas-counseling…"*
	GRRH counseling	*"…counseling from GRRH (still go there now)…"*
	Caritas and PACTA meetings	*"…attending AA-meetings…"; "…Caritas and PACTA meetings…"*
	Having a place to turn to	*"…you have a place to turn to…"*
	Support from sponsor	*"…M., his sponsor, whenever he feels depressed he talks to him…"*
	GRRH medication	*"…access to free medication in the hospital…"*
Social support	Financial support	*"…friends gave me money when in need…"*
	Support regarding work	*"…support in work…"*
	Support from social environment	*"…support from elders…"; "…good friends who support me in discussions…"*
Positive reactions from social environment	Positive change in attitude towards participant	*"…there was respect for him anywhere he moved…"; "…my wife changed and she started showing me love and treating me well as a result of my being in PACTA…"; "…friends started trusting me…"*
	Improved family life	*"…relationship with children has improved…"; "…there is good relationship with my partner…"*
	Improved community life	*"…relationship with neighbors has improved…"*
Experiencing success	Satisfaction with work life	*"…the working environment is good and […]…"*

	Ability to work	*"…I was able to do work…"; "…reducing increased my level of energy to work…"*
	Success in taking care of family	*"…I can ably support my family…"*
	Saving money	*"…can save and buy personal needs such as soap…"*
	Being a responsible person	*"…I have become responsible…"*
	Seeing progress	*"…see that he is moving ahead…"*
Visualizing future aims	Wish to improve family life	*"…had bad relationship with family and had to improve…"*
	Wish to successfully take care of the family	*"…looking for money to support my family…"*
	Future plans	*"…thinking of making more and more handcrafts…"*
	Wish to gain respect from others	*"…I wanted people to respect me…"*
Conditions supporting sobriety	Disliking the kinds of drinks in new surrounding	*"…in K. [name of an area] the drinks are different, he doesn't like it…"*
	Unfavorable environment	*"…K. [name of an area], conditions are hard there…"*
	Current health concerns	*"…her chronic disease: heart pressure, drinking is bad…"*
	Death of a close person	*"…loss of relatives…"*

Supplementary Table 5

Elements and aspects that were indicated at the 5-month-follow-up as having been not helpful in the Treatment Camp inpatient phase concerning the aim to quit drinking alcohol (code) clustered in broader components (category)

Category	Code	Representative direct quotes
Side-effects of medication	Side-effects of medication	*“…pills made people sleep…”*
	Side-effects of medication prevented understanding	*“…sleep: hard to understand the teachings…”*
Distraction by bodily concerns	Weakness	*„…people become weak…“*
	Pain in body	*“…you sit in a chair all the time and your body pains…”*
	Persistent craving	*“…I could not do without alcohol…”*
Components identified as lacking	No physical therapy	*“…no time for physical therapy (was nutrition and theoretical only)…”*
	No games and exercises	*“…games and exercises would have been nice…”*
	Food issues	*“…feedings: were not good, only very little milk…”*
	Lack of certificates	*“…because by giving that I could read it [the certificate] all the time I feel like quitting alcohol…”*
Duration was perceived as too short	Short duration	*“…period was too short…”*
Rule-breaking by fellow participants	Irritation by negative examples of fellow participants	*“…some participants had sachets [usually containing 100ml of gin (40%)] in their pockets…”*
Difficulties in understanding the information	Difficulties understanding the psychoeducation	*“…teaching about effects on body were better in GRRH (in the Treatment Camp she didn't understand them)…”*
	Too little learning material	*“…maybe they should display more pictures on the wall…”*
Unconvincing information and effects	Misinformation about effects of medication	*“…the way they […] made us think the medicine would help us stop…”*
	Medication did not work as intended	*“…I still get the feeling to drink, the urge, even after taking the medicine…”*
	Facilitators made quitting sound easy	*“…the way they made it sound easy…”*
	Information lacked justification	*“…some just mentioned things without giving reasons…”*
Unconvincing counselors	Under-qualified facilitators	*“…some counsellors lack experience in what they told us at the training…”*
	Inappropriate counselor matching	*“…it felt bad for me to be counseled by a very young lady…”*

	Formerly addicted facilitators as role models were not perceived effective	*"…some presenters were only showing they were out of addiction, but not pull people out of addiction…"*
	Lack of own experiences with alcohol-related problems in facilitators	*"…not very appealing, someone who gives own testimony is more lively…"*
Offending communication or attitude by counselors	Harsh language of facilitators	*"…one of the facilitators was not kind but rude on participants…"*
	Blaming by facilitators	*"…blaming…"*
	Disrespectful facilitators	*"…some organizers wanted to shout at people - no respect to participants…"*
	Participants were made to feel like a burden	*"…organizers tended to make people feel as if clients were a burden…"*
	Stigmatization	*"…stigmatization…"*
Internal barriers that prevented full engagement with the Treatment Camp	Participants developed resistance	*"…facilitator being rude makes me think that I can't take him seriously…"; "…people developed resistance…"*
	Participants felt shy	*"…some were quite rough and were blaming too much (one person in particular) […] made you feel shy…"*
	Participants remained stuck in self-blame	*"…self-blame remains there…"*
	Drinking to forget worries and hardship	*"…drink to forget worries or hardships…"*
	Missing friends	*"…no friends to console me…"*
Unfavorable reactions from outside social environment	Mockery from friends	*"…friends said I was taking drugs for the mad people…"*

Note. Fifty-two percent reported that they found nothing at all was not ideal or not helpful in the *Treatment Camp.*

Supplementary Table 6

Elements and aspects that were indicated at the 5- and 12-month-follow-up as having been not helpful after the Treatment Camp inpatient phase concerning the aim to quit drinking alcohol (code) clustered in broader components (category)

Category	Code	Representative direct quotes
Mishaps in aftercare	Failing to get medication	*"…tried to get medication but M.G. was not there…"*
	Failing to join AA-group	*"…AA-meetings: people didn't come…"*
Challenges to medication-compliance	No belief in medication	*"…do not believe in taking drugs from hospital…"*
	Fear of becoming addicted to medication	*"…[drugs from hospital] - another addiction…"*
	Medication did not work as intended	*"…the medicine never took away my urge to drink…"*
Content of the Treatment Camp was not sufficient	Advice did not suffice to make participant quit	*"…advices were not enough for me to quit…"*
Bodily states triggering urge to drink	Craving	*"…some urge to drink…"*
	Sleeping problems when sober	*"…if I do not drink alcohol I don't sleep…"*
	Physical pain	*"…Pain as a result of accident…"*
	Feeling weak	*"…Feeling weak…"*
	Feeling tired	*"…get very tired at place of work…"*
	Alcohol relaxes	*"…for relaxation…"*
Internal states triggering the urge to drink	Low self-esteem	*"… lowered self-esteem…"; "…failure in development of family unlike my brothers…"*
	Seeing no purpose in life	*"…resign purpose in life…"*
	Loneliness	*"…went back to drink to have company…"; "…isolated…"*
	Being bored	*"…to do away with boredom…"; "…boredom forces me to live with friends who drink…"*
External conditions triggering the urge to drink	Conflict	*"…feeling sad as a result of conflict with my brother…"; "…conflict with partner, why I sipped twice alcohol…"*
	Being unemployed	*"…no job - I was redundant…"*
	Homelessness	*"…I had no place to stay…"*
	Death of a close person	*"…grieving for my uncle*
	Having a hard time	*"…hard time…"*

	Tempting physical environment	*"…the environment that I live in…"; "…the campaigners give money to groups for drinking…"*
	Inability to change environment	*"…I can't change the situation…"*
	Language barrier at new home	*"…language barrier is a problem…"*
Social pressure to drink	Tempting social environment	*"…other people would offer her drinks…"*
	Occasions where drinking is expected	*"…celebrating survival for car accident…"*
	Forced into drinking by family	*"…[family] forced me to go back and drink heavily…"*
Suffering from negative consequences that accompany stimulus control	Restricted life-style	*"…started minimizing my ways - keep a distance with bad groups…"*
	Ambivalence about leaving friends	*"…it's difficult to make a decision to leave a friend…"*
	Having to avoid old friends	*"…not being able to go back to old friends…"*
	Danger of losing friends	*"…trying to distance myself from the drunkards made me to almost lose friends…"*
	Hard to make new friends	*"…difficult to identify people with good personality…"*
Unfavorable reactions from social environment after changing drinking behavior	Despise from drinking family	*"…family despised me and forced me into drinking…"*
	Isolation by friends	*"…friends in our group isolated me…"*
	Loss of friends due to change	*"…loss of friends…"*
	Rejection from non-drinkers	*"…people who do not drink hated me…"*
	Stigmatization	*"…mockery from the society…"*
	Worsened community life	*"…and also life became difficult for me in the community but later on normalized…"*
	Becoming sober is not valued by social environment	*"...people not knowing value of quitting alcohol…"*

Note. Thirty-seven percent reported that they found nothing at all was not ideal or not helpful after the *Treatment Camp.*

Supplementary Table 7

Elements and aspects that were indicated at the 5- and 12-month-follow-up as having caused a (re)lapse (code) clustered in broader components (category)

Category	Code	Representative direct quotes
Bodily states triggered the (re)lapse	Craving	*"…craving…"*
	Sleeping problems when sober	*"…stay awake most of the time if I don't drink…"*
	Alcohol relaxes	*"…sometimes just to relax, not like before…"*
	Wish to relieve fatigue	*"…to relieve fatigue after long day's work…"*
	Wish to relieve cold	*"…weather situation: cold at night…"*
	Wish to relieve physical pain	*"…pain in the leg…"*
Internal states triggered the (re)lapse	Negative emotions	*"…to treat my grieving…"; "…isolation (people compare him with his brother a lot which bothers him)…"*
	Being bored	*"…just out of boredom…"*
	Wish to experience positive emotions	*"…so he feels like going to people to be with them, make jokes, drink with them...."; "… for leisure only…"*
	Wish to forget problems	*"…when I drink it quenches my thoughts…"; "… hard life…"; "…lack of improved financial status…"*
External conditions triggered the (re)lapse	Fear of losing friends	*"…fear of losing friends…"*
	Lifestyle	*"…kind of lifestyle he lives…"*
	Death of a close person	*"…death of my auntie, who was my caretaker…"*
	Tempting physical environment	*"…staying in an environment where there is too much alcohol…"*
	Poverty	*"…poverty…"; "sometimes I drink because of poverty…"*
Social pressure to drink	Work depends on drinking friends	*"…to get back old friends who gave me work…"*
	Occasions where drinking is expected	*"…my brothers and sisters had come from K. [city] so I wanted to enjoy and celebrate with them the happiness…"; "…the day was independence-day because we had a party and I was with friends who drink…"*
	Pressure by social environment	*"…peer pressure…"; "…whenever I would meet my old group of friends they would encourage me to just taste so I ended up tasting…"*
	Tempting social environment	*"…excitement while with friends…"; "…being near friends who take alcohol…"*

Supplementary Table 8

Elements and aspects that were indicated at the 5- and 12-month-follow-up as having caused sobriety or successful reduction in alcohol intake to a low and stable level (code) clustered in broader components (category)

Category	Code	Representative direct quotes
Noticing positive change related to alcohol	Losing interest in alcohol	*"…I am losing interest in taking alcohol…"*
Noticing positive change in oneself	Improved work performance	*"…to improve performance in my work (has improved)…"*
	Improved self-control	*"…I no longer drink because I must drink…"*
Intentional recall of content of the Treatment Camp	Content of Treatment Camp	*"…the lessons we got from PACTA made me realize that I should not get so rooted into alcohol…"*
	Adhering to the information to stop drinking gradually	*"…since I learnt that I should not stop taking alcohol all at once, so I thought I should take at least something little…"*
	Personal letter to addiction	*"...one of the facilitators who made me write a letter refusing to drink…"*
	Meditation/Prayers	*"…Serenity Prayer…"*
	Awareness of negative health effects of alcohol	*"…learning how alcohol can destroy the liver…"*
	Awareness of negative effects of alcohol	*"…seeing the badness of drinking at my age…"*
	Awareness of negative effects of alcohol on work	*"…and alcohol is for the reason I lost my job…"*
	Awareness of negative financial effects of alcohol	*"…thinking back of how alcohol used to make me spend a lot of money…"*
Keeping one's mind set on sobriety	Active decision	*"…Came to the camp voluntarily…"; "…I decided I did not want to drink anymore…"*
	Self-reflection	*"…I was aging and needed to change my lifestyle…"; "…I do not want to do again what I did in the past…"*
	Realizing negative effects of alcohol on other drinkers	*"…I also see how drunkards embarrass themselves and my friends…"*
	Questions during pre-assessment	*"…the kind of questions they asked me in the first interview here…"*
Stimulus control strategies	Changing friends	*"…changed the group of friends…"*

	Avoiding drinking friends	*"…I avoided the drinking group…"*
	Not knowing non-hazardous limit	*"…felt it was better not to drink at all because he wouldn't know the limit (What is "a little"?)…"*
	Reduced outings	*"…from place of work I go home straight now…"*
Distraction strategies	Being busy (work, hobbies, sports)	*"...running my project - farm project…"; "…busy schedule…"; "…responsibilities…"*
Exposure to alcohol environment	Self-exposure	*"…goes to the bar as a method of self-exposure…"*
Elements of professional aftercare	Caritas, PACTA, GRRH meetings	*"…AA-meetings…"; "especially teaching from GRRH…"*
	Medication	*"…the medication I received…"*
Social support	Support from social environment	*"…family support…"; "…been advised by people (neighbors, relatives)…"*
	Support from partner	*"…my wife: she contributed too much to that…"*
	Advice from other ex-drinkers	*"…and also teachings from my brother who was an alcoholic…"*
Visualizing future aims	Wish to keep dignity	*"…to keep my dignity…"*
	Wish to gain respect from others	*"…to win my family's respect for me…"*
	Future plans	*"…and I want to do developmental thing for my future…"*
	Engagement to improve finances	*"…engaged myself to look for money…"*
Health conditions supporting sobriety	Physical disease	*"…ulcers disease, was quite serious…"; "…I am always sick…"*
	Feeling tired	*"…fatigue…"*
	Disability	*"…my disability…"*
External conditions supporting sobriety	Need for sobriety at work	*"…demand from the new job…"*
	Being unemployed	*"…no job…"*
	Having a new job	*"…new job…"*
	Death of a close person	*"…I lost many relatives since January…"*
	Possibility to keep old friends	*"…relieved him, so he could maintain his friends…"*
	Absence of drinking friends	*"…absence from peers – most of them in G. [city]…"*
	Poverty	*"…poverty / poor feeding…"*

Supplementary Table 9

Contents of the Treatment Camp that participants remembered at the 5-month-follow-up (code) clustered in broader treatment components (category)

Category	Code
Basic detoxification components	Sobriety
	Medication
	Healthy nutrition
	Decent accommodation
Imparting of knowledge on alcohol, AUD and its negative consequences	Psychoeducation
	Psychoeducation about types of alcohol
	Psychoeducation on drinking stages
	Psychoeducation about withdrawal
	Psychoeducation on negative effects of alcohol
	Stressing negative emotional consequences of alcoholism
	Psychoeducation via movies
Imparting of knowledge on the negative consequences of tobacco use	Psychoeducation on negative effects of smoking
Imparting of knowledge on how to overcome AUD	Psychoeducation on strategies for quitting
	Psychoeducation on self-control strategies
	Psychoeducation on positive social behavior
	Stressing the importance of quitting
	Teaching on how to make plans for a sober future
Spirituality/relaxation	Meditation/Prayers
Interactive single and group exchange on the impact of alcohol on the lives of participants	Counseling
	Testimonials
	Art Therapy
Basic counseling attitude	Free interaction
Undetermined social support	Sharing with others
	Social connection
Planning of aftercare	Offers of further help
